# The prognostic significance of the Birmingham Vasculitis Activity Score (BVAS) with systemic vasculitis patients transferred to the intensive care unit (ICU)

**DOI:** 10.1097/MD.0000000000005506

**Published:** 2016-12-02

**Authors:** Federico Biscetti, Angela Carbonella, Federico Parisi, Silvia Laura Bosello, Franco Schiavon, Roberto Padoan, Elisa Gremese, Gianfranco Ferraccioli

**Affiliations:** a Division of Rheumatology, Institute of Rheumatology, Fondazione Policlinico Universitario Agostino Gemelli, Catholic University School of Medicine, Rome; b Operative Unit of Rheumatology, Department of Internal Medicine, University of Padua, Padua, Italy.

**Keywords:** biomarkers, Birmingham Vasculitis Activity Score (BVAS), intensive care, vasculitides

## Abstract

Systemic vasculitides represent a heterogeneous group of diseases that share clinical features including respiratory distress, renal dysfunction, and neurologic disorders. These diseases may often cause life-threatening complications requiring admission to an intensive care unit (ICU). The aim of the study was to evaluate the validity and responsiveness of Birmingham Vasculitis Activity Score (BVAS) score to predict survival in patients with systemic vasculitides admitted to ICU.

A retrospective study was carried out from 2004 to 2014 in 18 patients with systemic vasculitis admitted to 2 different Rheumatology divisions and transferred to ICU due to clinical worsening, with a length of stay beyond 24 hours. We found that ICU mortality was significantly associated with higher BVAS scores performed in the ward (*P* = 0.01) and at the admission in ICU (*P* = 0.01), regardless of the value of Acute Physiology And Chronic Health Evaluation (APACHE II) scores (*P* = 0.50). We used receiver-operator characteristic (ROC) curve analysis to evaluate the possible cutoff value for the BVAS in the ward and in ICU and we found that a BVAS > 8 in the ward and that a BVAS > 10 in ICU might be a useful tool to predict in-ICU mortality.

BVAS appears to be an excellent tool for assessing ICU mortality risk of systemic vasculitides patients admitted to specialty departments. Our experience has shown that performing the assessment at admission to the ward is more important than determining the evaluation before the clinical aggravation causing the transfer to ICU.

## 1 Introduction

Systemic vasculitides are a group of diseases characterized by inflammation of the vessel wall. These clinical conditions are often severe, occasionally fatal, needing rapid diagnosis, and treatment. The clinical spectrum is extremely variable and the disease can occur both with nonspecific symptoms, such as expression of systemic inflammation (e.g., asthenia, fever, weight loss, arthralgia) and/or with specific symptoms or signs of organ dysfunction, such as respiratory distress, renal dysfunction, gastrointestinal, and neurologic disorders. The clinical course of systemic vasculitis can be characterized by severe flare of disease that may require admission to the intensive care unit (ICU), about 42% of patients with necrotizing vasculitis received diagnosis upon admission to ICU.^[1]^

Patients with antineutrophil cytoplasmic antibody (ANCA)-associated vasculitis have, if not treated, a poor prognosis with a mortality rate of 90% within 2 years of diagnosis.^[2]^ Granulomatosis with polyangiitis (GPA) is characterized by a mortality of 20.8% at 6.4 years.^[3]^ In eosinophilic granulomatosis with polyangiitis (EGPA) survival rates is 88.9% at 5 years.^[4]^ The prognosis of polyarteritis nodosa (PAN) is severe in patients not treated with corticosteroids with a survival rate at 5 years of 12% to 13% compared to the 48% to 53% of treated patients.^[5,6]^ For the microscopic polyangiitis (MPA) the survival rate, in treated patients, is 74% at 5 years.^[7]^ An international, multicenter, prospectively study was conducted by the European Vasculitis Study group to evaluate the long-term patient survival in ANCA-associated vasculitis. Deaths were recorded in about 25% of patients (133 out of 535 patients). The mortality ratio with respect to the general population was 2.6. Infections and vasculitis (respectively 48% and 19%) were the major causes of death during the first year of follow-up, while cardiovascular disease (26%), malignancy (22%), and infection (20%) were the main causes of death after the first year. Moreover patients with a severe disease revealed by a glomerular filtration rate < 15 mL/min and higher Birmingham Vasculitis Activity Score (BVAS) and elderly patients, with lower hemoglobin and higher white cell count showed a worse prognosis.^[8]^

The aim of this study was to shed light on patients with systemic vasculitides admitted to ICU and to identify possible prognostic biomarkers.

## 2 Methods

All investigations were approved by the A. Gemelli University Hospital Ethical Committee. This retrospective study was carried out from 2004 to 2014 at the Catholic University School of Medicine of Rome and at the University of Padua, Italy. All adult patients with systemic vasculitis admitted to both Rheumatology divisions and transferred to ICU due to clinical worsening, with a length of stay of over 24 hours, were evaluated in the analysis.

### 2.1 Patients

Eighteen patients (10 women and 8 men) with systemic vasculitis were included in the study. The diagnosis was made according to the 2012 Revised International Chapel Hill Consensus Conference.^[9]^ At the admission in the Rheumatology units, the BVAS was calculated. BVAS is a composite score made of 59 items organized into 9 different groups, expressing possible organ involvement. For every single item a score is set: the higher the global score achieved, the more severe the disease.^[10,11]^ All the patients were then transferred to ICU because of clinical complications. We selected only patients admitted to the Rheumatology unit due to vasculitis manifestations and then transferred to ICU, irrespective to the clinical complications. Upon admission to ICU, the BVAS and the APACHE II scores were calculated. The APACHE II scoring system is a systemic global score in which 13 parameters (expressing the clinical and organic status) are organized in a different range, to each of which a value is assigned. The global count is then related to the disease severity and to the mortality risk.^[12]^

According to ICU mortality, patients were divided in to 2 groups (survivors and nonsurvivors) and compared, in order to identify the predictive factors of outcome.

We compared this cohort to 16 control patients (10 women and 6 men), admitted to ICU with several clinical status and comorbidities. The control ICU patients were matched to cases by age and comorbidities.

### 2.2 Statistical analysis

Analysis was performed with the statistical software package SPSS 20 (SPSS, Inc., Chicago, Ill., USA). A descriptive analysis was first performed. The univariate analysis was performed using 2-tailed Mann–Whitney test for quantitative variables and Pearson Chi^2^ test or Fisher exact test for qualitative variables when appropriate. Multivariate analysis was performed by logistic regression in order to evaluate the adjusted effects of the different variables. The Kaplan–Meier methodology was used to generate the long-term survival curve. A value of *P* < 0.05 was considered as significant.

## 3 Results

The vasculitis cohort included 8 patients with GPA, 5 with MPA, 2 with Takayasu, 1 with EGPA, 1 with cryoglobulinemia, and 1 with Intestinal and pulmonary vasculitis. Global demographic parameters, comorbidities, and medical history are shown in Table 1. The vasculitis population was composed of 8 men (44.5%) and 10 women (55.5%). The average age was 62.0 years, while the average duration of disease was 5.76 years. Out of 18 patients, all of them received steroid therapy, and 12 (66.7%) had previously received immunosuppressive therapy: 6 (33.3%) received cyclophosphamide (CYC), 7 (38.8%) azathioprine (AZA), and 2 (11.1%) methotrexate (MTX). None of the considered patients received biological therapy.

**Table 1:**
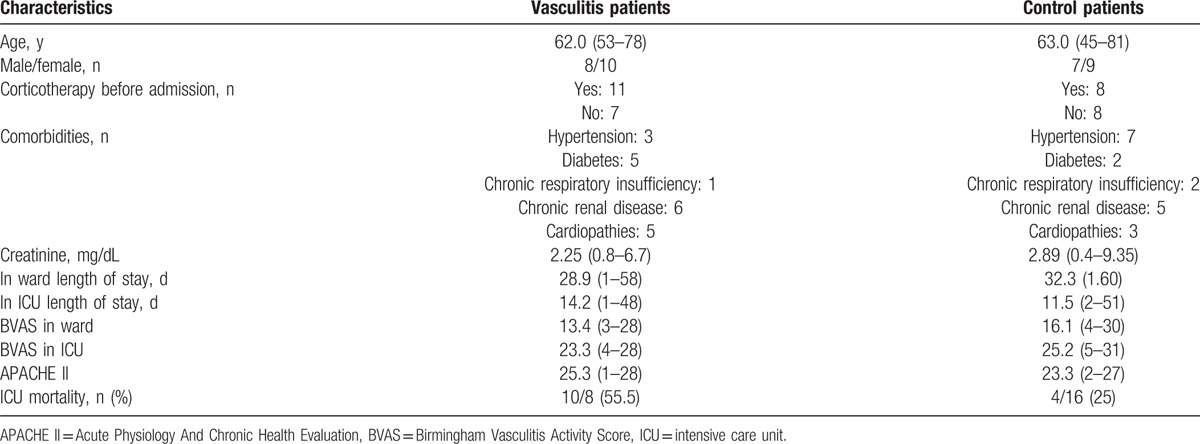
Patients characteristics.

Causes of admission to ICU for patients with vasculitis were different: 9 (50.0%) sepsis, 3 (16.6%) respiratory failure, 2 (11.1%) cardiac shock, 2 (11.1%) hospital-acquired pneumonia, 1 (5.55%) diffuse alveolar hemorrhage, 1 (5.55%) spontaneous hemorrhage. In septic patients, microbiological test and blood culture identified methicillin-resistant *Staphylococcus aureus* (MRSA) in 3 (16.6%), *Staphylococcus epidermidis* in 1 patient (5.55%), *Escherichia coli* in another one (5.55%), and *Klebsiella pneumoniae* in the last one (5.55%); in the remaining 3 patients with sepsis, the pathogen was not identified. Out of 18 patients, 8 (44.4%) survived, while 10 (55.6%) died in ICU. Therapeutic interventions adopted in ICU are shown in Table 2.

**Table 2:**
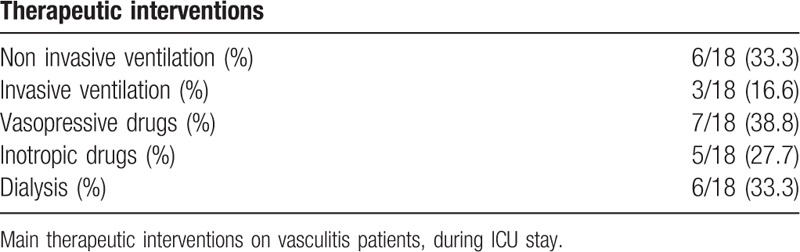
Interventions.

The control patients were admitted to ICU for ventricular tachyarrhythmia (4/16), pulmonary edema (3/16), cardiogenic shock (3/16), septic shock (3/16), myocarditis (2/16), acute respiratory failure (1/16). Out of 16 patients, 6 (37.5%) died in ICU, while 12 (75.5%) survived.

**Figure 1:**
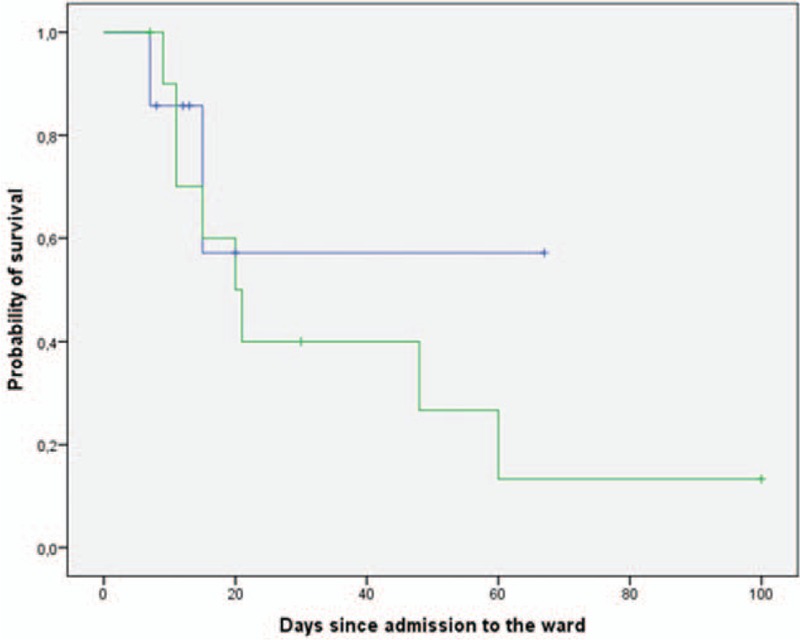
Kaplan–Meier curve for 100 days survival after the ward admission of patients with vasculitis diseases. Patients with initial BVAS > 8 are indicated in green and patients with initial BVAS < 8 are indicated in blue.

As shown in Table 3, patients who died in ICU presented a higher BVAS value, revealing a more aggressive vasculitis compared to the other ones. As shown in Table 3, at univariate analysis comparing survivors and nonsurvivors, ICU mortality rate was significantly associated with higher BVAS scores (*P* = 0.01). Deceased patients had higher creatinine values (*P* = 0.06) and lower values of hemoglobin (*P* = 0.09) (data not shown). Interestingly, the APACHE II score was statistically similar in survivor group and in nonsurvivors (*P* = 0.50). No variables associated with mortality in univariate analysis remained significant in the multivariate analysis. We used receiver-operator characteristic (ROC) curve analysis to evaluate the possible cutoff value for the BVAS in the ward and we found that a BVAS in the ward >8 and a BVAS in ICU >10 might be a useful tool to predict in-ICU mortality (Table 4, Fig. 1).

**Table 3:**
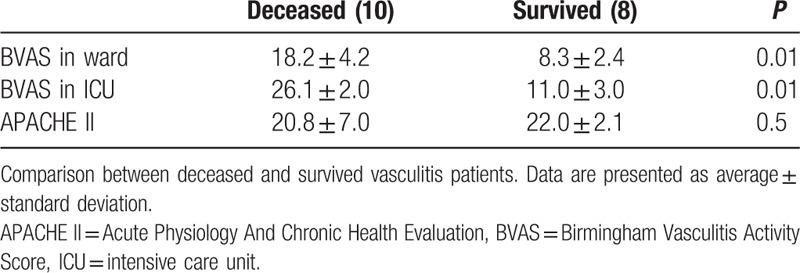
BVAS and APACHE II comparison.

**Table 4:**
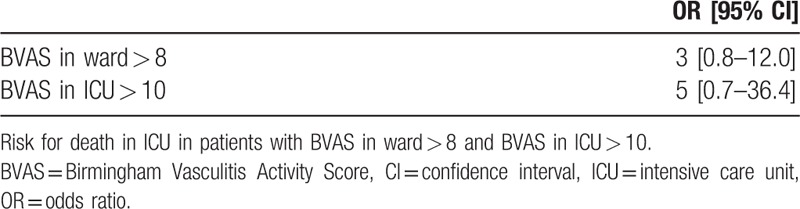
Risk for death.

Considering the entire cohort of 34 patients (cases and controls), 14/34 (41.2%) died in ICU. There was no statistical difference in the average of creatinine and hemoglobin between the 2 groups.

## 4 Discussion

Systemic vasculitides represent a heterogeneous group of diseases that share clinical features including fever, arthralgia, respiratory distress, renal dysfunction, and neurologic disorders. These diseases may often cause life-threatening complications requiring admission to ICU^[13,14]^ depending on several factors: the exposure to external agents, mostly infections, the therapy, which usually results in immunosuppression, and the comorbidities of the patient.^[13,15]^

In an analysis of over 1200 studies, systemic vasculitides were found to be the third cause among all autoimmune diseases that led to admission to ICU, with a percentage of 15% of the total 203 patients, mainly for respiratory failure, vasculitis relapse, and infectious complications. The causes of transfer to ICU among patients with systemic vasculitis were: respiratory failure, followed by reactivation of the vasculitis, and infectious complications. The mortality rate of vasculitis patients in ICU ranged from 10% to 33%.^[16]^ Similarly, a previous study found that, among the systemic autoimmune diseases, the necrotizing vasculitides often led to hospitalization in ICU, right after the systemic lupus erythematosus (SLE).^[17]^

In 2006, Khan and coworkers described the course and prognostic factors of 38 patients with small-vessel vasculitis admitted to the ICU. To assess disease activity they used the Acute Physiology And Chronic Health Evaluation (APACHE) III score, the Sequential Organ Failure Assessment (SOFA) score and the Birmingham Vasculitis Activity Score for Wegener granulomatosis (BVAS/WG).^[18]^ The APACHE is a simple and accurate assessment scale of the severity of disease in critically ill patients newly admitted to ICU^[12]^ and it is a mortality prediction score useful to define the risk stratification. The SOFA is a score used to asses organ dysfunctions in patients with sepsis: it considers respiratory, coagulative, liver, cardiovascular, central nervous system (CNS), and renal parameters,^[19]^ and it is closely related to mortality. The BVAS/WG is a score designed to measure the level of disease activity, by identifying all the possible organ clinical manifestations. In their series the most represented vasculitis was GPA followed by MPA. The causes of admission to ICU were diffuse alveolar hemorrhage (37%), sepsis (13%), seizure (8%), and pneumonia (5%). Septic shock was the leading cause of death. The mortality rate of their patients was lower than expected: APACHE III-predicted mortality rate was 25.7%, the 28-day mortality rate was 11%. Moreover, between surviving and nonsurviving patients there were differences in APACHE III and SOFA scores, but not in BVAS score.^[18]^

More recently Befort and colleagues evaluated the outcome of 31 systemic vasculitis patients admitted to ICU. They found that GPA was the vasculitis that most frequently led to ICU admission; clinical manifestation of active vasculitis was the cause for transfer to ICU, followed by septic shock. In ICU, 52% of patients died and BVAS score and Simplified Acute Physiology Score (SAPS) II were the parameters predicting mortality in a multivariate analysis.^[14]^

Similarly, Cruz and coworkers reported in a retrospective study that active systemic necrotizing vasculitis was the most frequent reason of ICU admission. Moreover, in 42% of the 210 patients that they reviewed, the vasculitis was first diagnosed precisely in ICU as its first clinical manifestation was so aggressive as to require ICU admission. The second cause leading patients to ICU was infection. APACHE II and SAPS II scores at ICU admission were associated with mortality during the hospitalization. On the other hand, BVAS score predicted the long-term prognosis at the end of follow-up (about 30 months).^[1]^

Different data were reported by Burkhardt that retrospectively analyzed 17 patients with GPA admitted to ICU: severe hemoptysis was the first cause for the admission followed by respiratory failure. They found that APACHE II score >24 and a period of time in ICU >10 days were associated with death.^[20]^

Searching for biomarkers as possible tools for outcome assessment in chronic diseases has become an interesting and fashionable topic in clinical and basic research and in clinical practice, not only in rheumatology, but also in other clinical areas such as hematology and oncology. Moreover, biomarkers with well-evaluated clinical relevance could be used as surrogate endpoints in clinical trials.^[21]^

Biomarkers of disease activity and severity and their role in prediction the evolution of the disease over time were sought with the aim to improve the management of systemic vasculitides. To date, the predictive power of ANCA-antibody titer increasing in terms of disease relapses are controversial, although ANCA positivity is frequently necessary to make a diagnosis. A wide variation from 24% to 100% of serial ANCA measurements has been reported for monitoring disease activity and for predicting disease relapses.^[22]^ Recently, Monach identified 3 biomarkers to distinguish remission from active disease in ANCA-associated vasculitis. The C-X-C motif chemokine 13 (CXCL13) also known as B lymphocyte chemoattractant (BLC), the matrix metalloproteinase-3 (MMP-3), and TIMP metallopeptidase inhibitor 1 (TIMP-1) are the best-performing markers in discriminating active disease from remission, even if compared with reactive protein C (CPR) and erythrocyte sedimentation rate (ESR).^[23]^ Additional potential biomarkers in patients with vasculitides are represented by monocyte chemoattractant protein-1 (MCP-1),^[24]^ the von Willebrand factor antigen,^[25]^ the Pentraxin-3 (PTX3),^[26]^ and the Eotaxin-3.^[27,28]^ A summary of the main studies focused on possible biomarkers in patients with vasculitides is reported in Table 5.

**Table 5:**
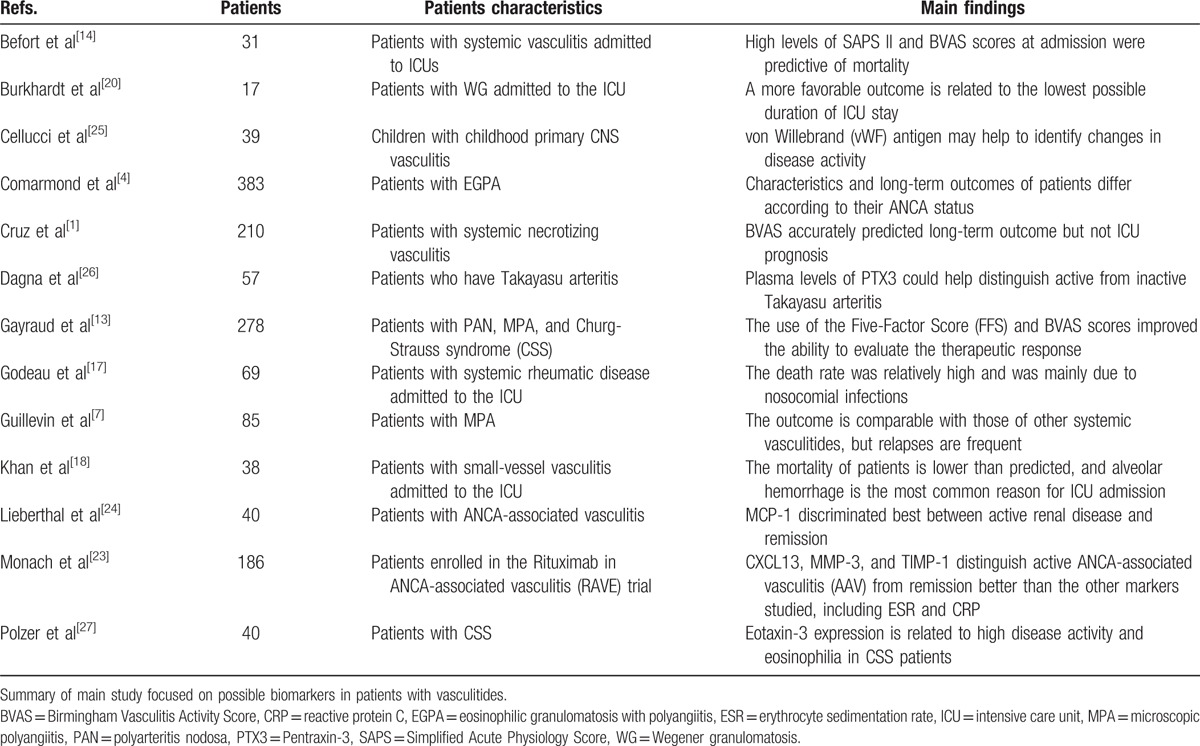
Study focused on possible biomarkers in vasculitis patients.

Finally, procalcitonin (PCT) could be a challenging biomarker to distinguish infectious from active inflammatory disease. Its circulating serum levels are increased during infection while they are normal during vasculitis disease such as ANCA-associated vasculitis and giant cell arteritis.^[29–31]^

Nevertheless, satisfactory and definitive results have not been obtained regarding candidate molecules and scores to predict the outcome of patients with vasculitis admitted to ICU. Therefore, we focused our attention on the BVAS and on the APACHE II scoring systems.^[11,12]^ In fact, surrogate markers are very useful to allow the clinician to stratify the risk regarding the patient as soon hospitalized, and the BVAS score represents the ideal, among those currently available, to evaluate the patient with systemic vasculitis. The BVAS is also used as a prognostic tool, although the predictive power is not yet defined. Specifically, there is evidence showing that this score correlates quite well with the long-term outcomes of patients admitted to ICU, but that it is not reliable in predicting mortality.^[1]^ In contrast, the latest data demonstrated that BVAS score is independently related to the mortality in ICU.^[14]^ Starting from these reports, we stratified our patients according to the value of the BVAS at the admission to the ward and to ICU. Therefore, we have also performed the APACHE II score in our study. We found that the BVAS in ICU correlated with the mortality of the patient. It is important to note that the BVAS was not originally designed for patients admitted to the intensive care and with life-threatening complications. In fact, the type of items taken into account may not be completely applicable to this kind of patient and may not reflect the actual severity of the clinical situation. The APACHE II score is designed to be applied within the first 24 hours after the admission to ICU and it is more reliable in the assessment of patients. In particular, the APACHE II score depends on factors that influence outcome in ICU patients (e.g., chronic diseases, patient reserve and severity of acute illness). Conversely, the BVAS is more related to the degree of single organ dysfunction due to acute illness. Interestingly, we found no statistically significant difference between vasculitis patients who survived and those who died, regarding this scoring system. Notably, when we have considered the entire cohort of patients, with or without vasculitis, the APACHE II score was higher in patients deceased in ICU. A possible interpretation could be that the APACHE II is not a score designed for patients with specific diseases such as systemic vasculitis. For this reason, we have chosen to test the diagnostic power of the BVAS, the score specifically conceived for patients with vasculitis, in the ideal conditions for the evaluation, that is, the beginning of hospitalization. We found that the BVAS score was significantly higher in the nonsurvivor group of patients, demonstrating the relationship between the BVAS evaluation in the ward and the mortality in ICU.

There are several limitations in our study. First, given the small number of patients, a statistically significant difference is not evident among the other parameters. In fact, we observed a tendency to the difference regarding the kidney function between survivors and nonsurvivors. Obviously, renal involvement depends on type of vasculitis and on the staging. In addition, the small number of patients probably resulted in the inability to obtain significant data from the multivariate analysis. In addition, we did not obtained stratified patients according to steroid pulse therapy, a commonly used regimen for life-threatening vasculitis. Furthermore, the most important cause of transfer to ICU in our experience was infections, and in particular the sepsis. Given the small number of patients, it was not possible to isolate the infectious event in the confounding factors. The widely recognized biomarker for the septic condition is the PCT. In fact, PCT has been demonstrated to be the best marker for differentiating patients with sepsis from those with systemic inflammatory diseases.^[32,33]^ However, we have not taken in account the PCT levels, because the PCT measurements were not available for an adequate number of patients in our cohort. Finally, as a retrospective study, also significant results should be considered with caution. However, these data represent an interesting starting point for further studies and confirm the importance of clinical assessment through the BVAS scoring system at the admission to the Rheumatology unit, rather than after the transfer to ICU.

In conclusion, the BVAS is an excellent tool for assessing the in-ICU mortality risk of patients with systemic vasculitis admitted to specialist departments. Our experience has shown that it is more important that the assessment be carried out at admission to the ward, even before the clinical aggravation determines the transfer to ICU. The APACHE II score appears not a good prognostic predictor for these peculiar patients.
